# Luminescence of In(III)Cl-etioporphyrin-I

**DOI:** 10.3390/ijms242015168

**Published:** 2023-10-14

**Authors:** Andrey I. Koptyaev, Yuriy A. Zhabanov, Georgy L. Pakhomov, Piotr P. Pershukevich, Serguei M. Arabei, Pavel A. Stuzhin

**Affiliations:** 1 Faculty of Organic Chemistry and Technology, Ivanovo State University of Chemistry and Technology, 153000 Ivanovo, Russia; zhabanov@isuct.ru (Y.A.Z.);; 2 Institute for Physics of Microstructures of Russian Academy of Sciences, 603950 Nizhny Novgorod, Russia; 3 B.I. Stepanov Institute of Physics of the National Academy of Sciences of Belarus, 220072 Minsk, Belarus; p.persh@ifanbel.bas-net.by; 4 Agro-Power Faculty, Belarusian State Agrarian Technical University, 220012 Minsk, Belarus; serguei.arabei@gmail.com

**Keywords:** synthetic etioporphyrins, molecular structure, spectroscopy, fluorescence, phosphorescence, quantum yields

## Abstract

The luminescent and photophysical properties of the etioporphyrin-I complex with indium(III) chloride, InCl-EtioP-I were experimentally studied at room and liquid nitrogen temperatures in pure and mixed toluene solutions. At 77 K, in a 1:2 mixture of toluene with diethyl ether, the quantum yield of phosphorescence reaches 10.2%, while the duration of phosphorescence is 17 ms. At these conditions, the ratio of phosphorescence-to-fluorescence integral intensities is equal to 26.1, which is the highest for complexes of this type. At 298 K, the quantum yield of the singlet oxygen generation is maximal in pure toluene (81%). Quantum-chemical calculations of absorption and fluorescence spectra at temperatures of 77 K and 298 K qualitatively coincide with the experimental data. The InCl-EtioP-I compound will further be used as a photoresponsive material in thin-film optoelectronic devices.

## 1. Introduction

The luminescence of metalloporphyrins in solutions and various matrices has been studied in sufficient detail, and it has been found that spectral and photophysical properties substantially depend on both the nature of the peripheral substituents in the macroheterocyclic ligand and the central metal ion [1,2,3,4,5,6,7,8,9]. Most popular porphyrin molecules bear alkyl or pseudo-alkyl substituents in the β-positions of the pyrrole rings, which usually have a minor influence on the luminescence parameters but increase the solubility in common organic solvents.

Recently, our group began studies on etio-type porphyrins, synthetic analogs of naturally occurring petroporphyrins—components of fossil fuels. They showed good stability during high vacuum sublimation [10] and high photoconductivity in thin sublimed films [11], which makes them promising candidates for device applications [12,13]. Among various transition metal complexes, a new complex of etioporphyrin-I with indium(III) chloride stands out with its unusual 2D-layered packing motif of molecules in a crystal [12].

In photovoltaic converters with an active layer based on small-molecule semiconductors, where the intermolecular interaction is weak, charge generation proceeds through the absorption of a photon by a molecule and its transition to the excited state S_1_. The desired parameters of an efficient sensitizer for use in photoresponsive devices are a long lifetime of the triplet state T_1_, low quantum yield and a short lifetime of fluorescence [1,14]. On the other hand, it was noticed in [1] that advantageous is the predominant pathway of excitation energy relaxation from S_1_ to T_1_ state, which can be identified by the high quantum yield of singlet oxygen generation. All of these features can be reached by embedding of indium(III) into the chelate window, which facilitates intersystem crossing S_1_~>T_1_ (ISC) because increased the spin–orbital interaction partially removes spin forbiddance (internal heavy atom effect) [1,2,9]. 

**Figure 1:**
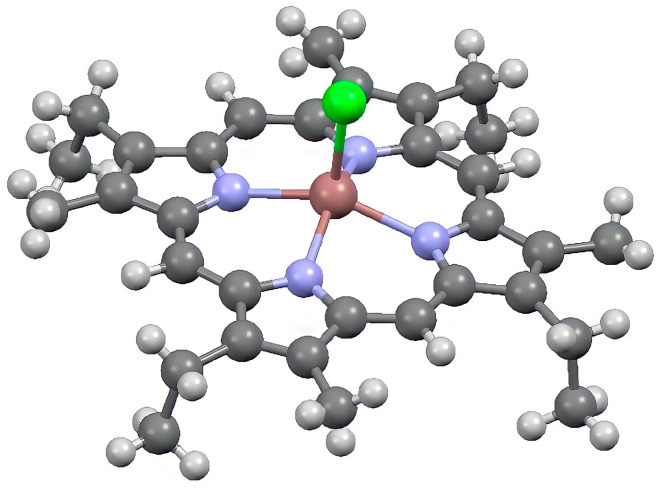
Molecular structure of InCl-EtioP-I determined from X-ray analysis [12].

Photoluminescence properties of metal-free etioporphyrins and some metal complexes of it were reported [3,4,5,6,7,15]. However, for an indium(III) complex, such experiments have not yet been performed [12]. In this work, we report experimental and theoretical estimates of luminescent and photophysical properties of InCl-EtioP-I (Figure 1) at 298 and 77 K in toluene solutions.

## 2. Results and Discussion

### 2.1. Absorption and Luminescence Spectra of InCl-EtioP-I

**Figure 2:**
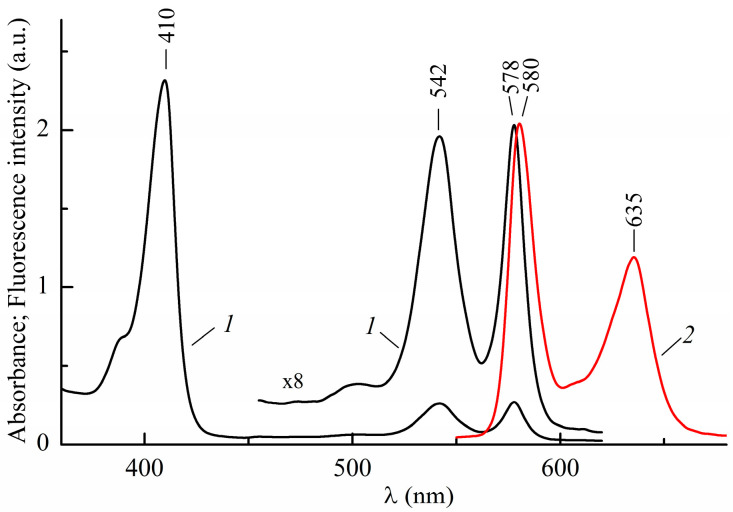
Room temperature absorption (1) and fluorescence spectrum at λ_ex_ = 409 nm (2) of InCl-EtioP-I in toluene.

The electronic absorption spectrum in toluene at 298 K is shown in Figure 2 (curve 1). It contains an intensive Soret-band at 410 nm and two weak bands in the middle part of the visible range at 542 and 578 nm [12]. According to the four-orbital Gouterman model [8,16], the long-wavelength band at 578 nm is due to electronic transitions to a doubly degenerate state of the metallo-porphyrin complex, resulting from an increase in symmetry, S_1,2_←S_0_. Similar situations suggest the Soret band that corresponds to transitions to a doubly degenerate state S_3_ and S_4_ for use. The band at 542 nm is of vibrational origin.

The fluorescence spectrum in toluene at 298 K (Figure 2, curve 2) has two bands with maxima at 580 and 635 nm. Here, a short-wavelength band is responsible for a purely electronic transition from a lower degenerate singlet excited state back to the S_0_-state, and a long-wavelength band at 635 nm is its vibrational satellite. The shape of the fluorescence spectrum of InCl-EtioP-I in toluene resembles fluorescence spectrum of Zn(II)-EtioP-I in benzene, which also shows an intense short-wavelength band at 572 nm [6]. The likely reason for this resemblance is that both central atoms have alike closed-shell electronic configurations. TD-DFT calculations from Ref. [17] show that spectra of Mg-porphin and Mg-EtioP-I are practically identical in the visible region, while the energies of electronic transitions for the Soret band are by 0.2–0.3 eV lower for Mg-EtioP-I than for MgP. This implies that four methyl and four ethyl substituents have little effect on the electronic spectra. Starting from this implication and taking into account the presence of one degenerate absorption band in the visible and one in the UV regions, we assume that the conjugated π-chromophore system of InCl-EtioP-I has an effective symmetry close to *D*_4h_. 

As can be seen in Figure 2, the mirror symmetry of the absorption spectrum with the fluorescence spectrum in the visible region is broken. The relative intensity of the vibronic band at 542 nm in the absorption spectrum is greater than that of the corresponding band at 635 nm in the fluorescence spectrum, whereas its vibronic frequency is less (~1150 cm^−1^ vs. ~1500 cm^−1^). Most likely, the violation of the mirror symmetry both in terms of intensities and frequencies is the result of electronic–vibrational interactions during the formation of vibronic bands. The degeneracy of electronic states in the Soret band located near the vibrationally induced states in the visible range (ΔE ≈ 6000 cm^−1^) increases the intensity of the corresponding vibronic bands due to the allowed transition to the level of the Soret band. The absence of mirror symmetry can also be associated with an increase in the nonplanarity of the tetrapyrrolic ligand (Figure 1) in the excited state, although a small Stokes shift (~60 cm^−1^) indicates the minor effect of such a change.

**Figure 3:**
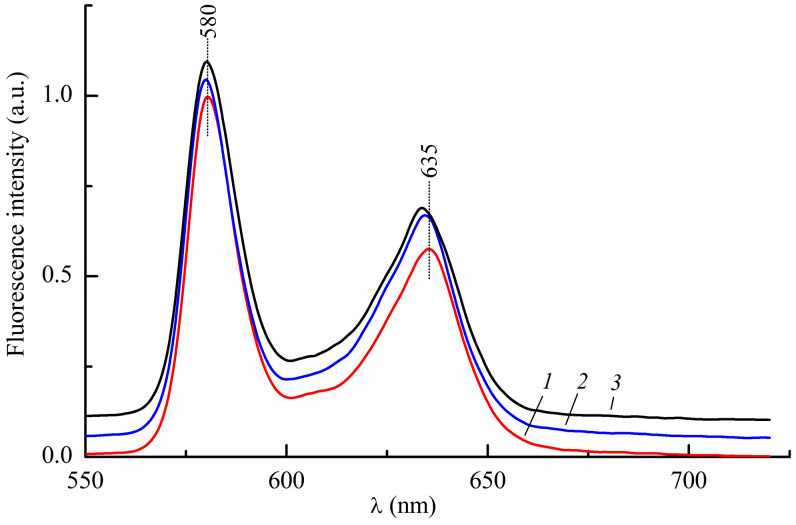
Room temperature fluorescence spectrum of InCl-EtioP-I at λ_ex_ = 409 nm in pure toluene (1), in a binary toluene:diethyl ether 1:2 solution (2) and in a ternary toluene:diethyl: methyl iodide 1:2:1 solution (3).

The absorption and fluorescence spectra of InCl-EtioP-I in mixtures of toluene with other solvents, such as diethyl ether or methyl iodide, do not practically differ from the spectra in pure toluene, except for a small hypsochromic shift within 1 nm. Figure 3 illustrates the effect of solvent on the fluorescence spectrum of InCl-EtioP-I at 298 K.

**Figure 4:**
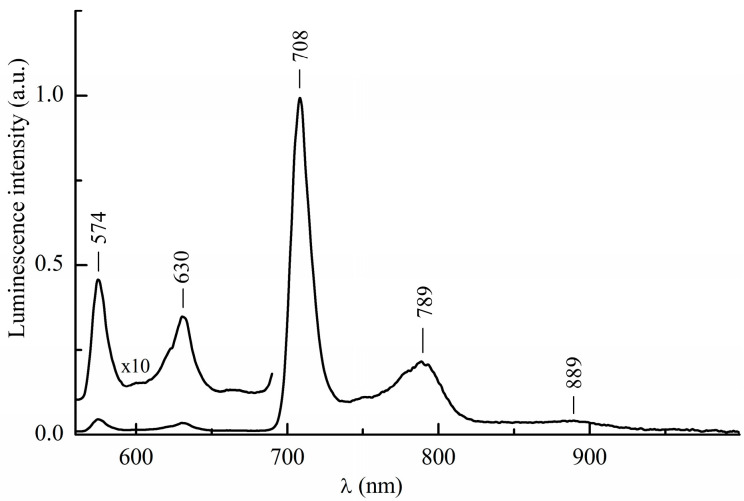
Liquid nitrogen luminescence spectrum of InCl-EtioP-I at λ_ex_ = 409 nm a binary toluene:diethyl ether 1:2 solution.

**Figure 5:**
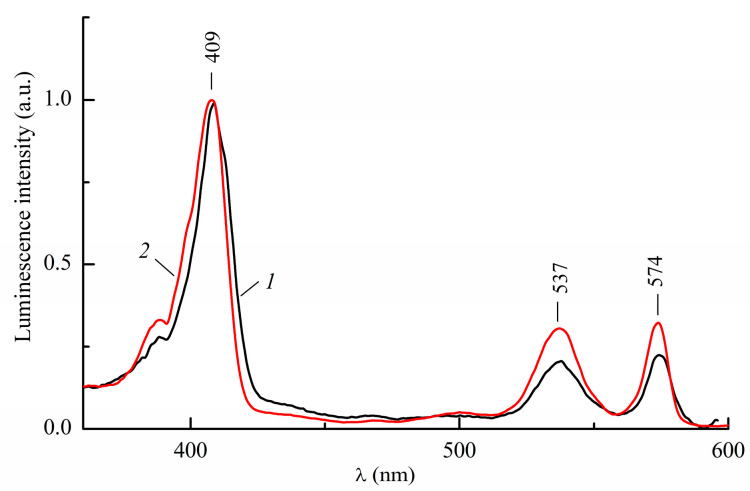
Liquid nitrogen fluorescence excitation spectrum, λ_reg_ = 630 nm (1) and phosphorescence excitation spectrum, λ_reg_ = 709 nm (2) of InCl-EtioP-I in a binary toluene:diethyl ether 1:2 solution.

The luminescence spectra at 77 K were obtained in the solvent mixtures that form a glassy matrix upon freezing (Figure 4). A decrease in temperature leads to a hypsochromic shift of the fluorescence bands by about 5 nm, which is consistent with the direction of the temperature shift of the spectral bands for most tetrapyrroles [18,19]. Next, an intense band at 708 nm emerges in the spectrum, accompanied by two descending long-wavelength satellites, as shown in Figure 4. The similitude of the luminescence excitation spectra recorded with registration wavelengths 630 and 709 nm shown in Figure 5 suggests that the new luminescence bands belong to emission from an InCl-EtioP-I molecule. Other photophysical measurements (see below) allow us to unambiguously attribute emission with wavelengths longer than 700 nm to phosphorescence. This agrees with the literature data on the position of the phosphorescence bands of etioporphyrin-I complexes with other metals [3,4,5,6].

The integral intensity of the phosphorescence of InCl-EtioP-I exceeds the integral intensity of the fluorescence by 26.1 times. For all known metal complexes of etioporphyrin-I, this ratio is either close to or much less than unity [3,4,5,20]. In Ref. [4] the fluorescence of metal-free etioporphyrin I and its complexes with Cu or VO at 77 K was observed not only for monomeric molecules, but also for their fluorescent aggregates in a frozen matrix, and the shape of the emission spectrum varied with the excitation wavelength. In the case of InCl-EtioP-I, no such trends have been identified. This fact, along with the similarity of the fluorescence excitation spectra in Figure 5, indicates that only single (monomeric) molecules, rather than dimers or other aggregates [4], participate in both types of emission. 

The phosphorescence spectrum in Figure 4 consists of three bands, with the frequency interval between two short-wavelength bands being equal to ~1450 cm^−1^, which is very close to the frequency interval of fluorescence bands. This again confirms that both types of emission belong to the same molecular centers. The equality of the abovementioned frequency intervals follows from the fact that the same vibrational frequencies belonging to the ground state appear both in the fluorescence and phosphorescence spectra. However, the phosphorescence spectrum differs by the intensity of the long-wavelength vibronic band at 789 nm, which is much lower than that of the band at 709 nm. In the fluorescence spectrum, the relative suppression of the vibronic satellite intensity is less pronounced. 

The observation of intense phosphorescence can be interpreted based on the existing concept of the spin–orbit interaction and mutual arrangement of the singlet S_1_ and triplet T_1_ levels in the porphyrin metallo-complexes [9]. In the case of the InCl-EtioP-I molecule, a heavy indium atom acts as a spin–orbit perturbing factor that strongly enhances the interaction between the triplet and singlet levels (ΔE_ST_ ≈ 3300 cm^−1^). This partly eliminates ISC forbiddance, and hence amplifies phosphorescence. The probability of ISC significantly exceeds the probabilities of radiative fluorescent S_1_→S_0_ and nonradiative S_1_~~>S_0_ transitions. Lowering the temperature leads to freezing of the oscillations, relaxation through which is a competing process of phosphorescence, and hence promotes its enhancement in any etioporphyrin [2,3]. Some shift of the luminescence bands with decreasing temperature is due to a decrease in the speed of rearrangement of the geometry of a molecule during its transition to an excited state [9]. 

### 2.2. DFT Calculations

**Figure 6:**
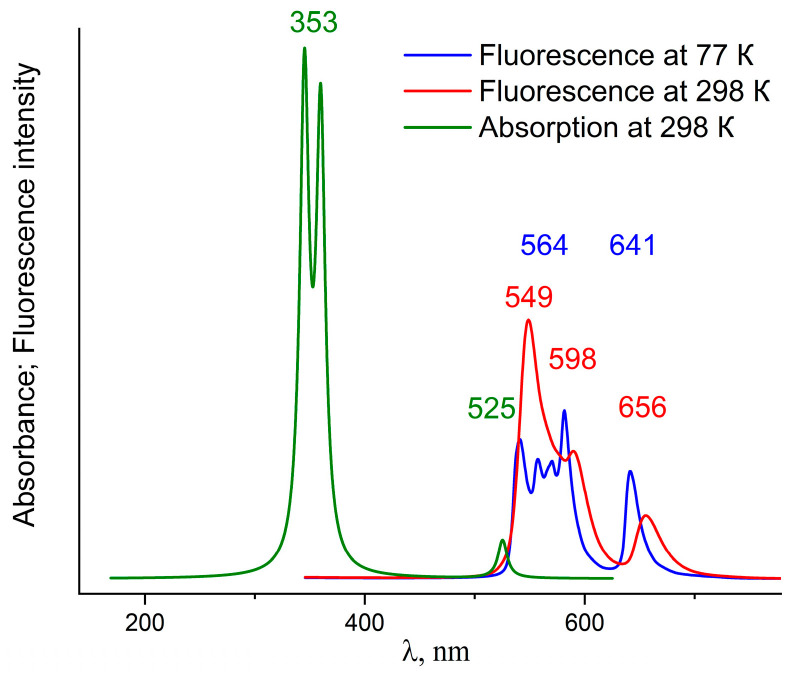
Calculated absorption (green curve) and fluorescence spectra of InCl-EtioP-I.

The electronic absorption spectrum and fluorescence spectra of InCl-EtioP-I at temperatures of 77 K and 298 K calculated using DFT are shown in Figure 6.

The interpretation of absorption bands is similar for metalloporphyrins [17,21,22] and relies on the Gouterman model [16,23]. The electronic transitions underlying the theoretical absorption spectrum of In-EtioP-I have been discussed in more detail earlier [12]. The theoretical fluorescence spectra were obtained using the doubly degenerate lower excited singlet state. Similarly to the experiment, in the theoretical fluorescence spectrum at 298 K the band at 549 nm corresponding to the transition between main vibronic states (S_1_→S_0_) have a higher intensity than vibronic bands at longer wavelength. In is interesting that in the theoretical spectrum at 298 K appearance of two vibronic bands is predicted at 590 and 656 nm (Figure 6, red curve). Presumably, the 590 nm band corresponds to an electronic transition to the vibrational sublevel, whose probability in the experimental spectrum is very low. 

All bands of the calculated fluorescence spectrum are shifted hypsochromically at 77 K, and the short-wavelength region contains four bands, three of which correspond to vibronic transitions, as shown in Figure 6 (blue curve). This is similar to what has been observed in experimental work [2] for metal complexes of TPP. According to DFT, probabilities of radiation (fluorescent) transitions *k*_F_ are 2.89 × 10^11^ s^−1^ and 4.14 × 10^11^ s^−1^ at 77 K and 298 K, respectively.

### 2.3. Photophysical Properties

As noticed above, the lifetimes of the singlet (τ_F_) and triplet (τ_P_) excited states are useful parameters in determining the efficiency of a molecular photoconductor, they help to roughly estimate the ratio of singlet-to-triplet excitons formed upon absorption of incoming photons, χ_S_ = N_S_/N_T_. Interestingly, the lifetime values considered “good” in photovoltaic devices are the same as for porphyrin compounds used in photodynamic cancer therapy, PDT [24]. In photovoltaics, it is desirable to obtain the molecule capable of producing a long-lived triplet exciton, i.e., χ_S_ < 0.01 [24], whereas material with a high yield of singlet excitons (χ_S_ ≥ 0.2) is rather suitable for use in organic light-emitting diodes [25]. In other words, the performance of a photodetector depends on the efficient generation of triplet excitons within the active layer, other relaxation pathways of the lowest excited singlet state, e.g., through fast radiative (fluorescence) or nonradiative (internal conversion) are unwanted because they reduce the probability of populating the T_1_-state.

To assess the prospects of InCl-EtioP-I molecule in this field, basic photophysical parameters (τ_F_, τ_P,_, fluorescence and phosphorescence quantum yields (φ_F_) and (φ_P_), singlet oxygen generation yield (φ_Δ_)) are collected in Table 1.

The fluorescence of InCl-EtioP-I follows an almost monoexponential decay only in pure toluene. In a mixture of toluene with diethyl ether, τ_F_ corresponds to the sum of two exponents with comparable contributions, while in a triple solution toluene:diethylether:methyl iodide the faster exponent dominates. Possibly, a change in the chemical composition of a medium leads to the formation of various solvate envelopes incorporating guest molecules around the InCl-EtioP-I complex. The solvent envelopes do not have a noticeable effect on the position of the energy levels in the porphyrin chromophore, since the absorption and luminescence spectra in different media are similar, but they are able to amend the ratio of the probabilities of electronic transitions between them. 

Since the phosphorescence decay strictly obeys monoexponential law indicating that all the media in question contain phosphorescent centers of the same type. The addition of diethyl ether or methyl iodide to a toluene solution does not affect the fluorescence intensity significantly, as shown in Table 1. Contrary to expectations, with the addition of methyl iodide the φ_P_ value decreases from 10.2% to 7.1%, i.e., observed external heavy-atom (here, iodine) effect is negative; τ_p_ decreases from 17.0 to 6.0 ms. The sign of the heavy atom effect caused by CH_3_I reflects the trade-off between two contributions to the spin–orbital interaction since the components of the matrix element of the spin–orbit interaction operator have opposite signs. The first contribution is made by the central indium atom (internal effect), and the second contribution is made by the iodine atoms of the solvent (external effect), so spin–orbit perturbations interfere destructively [9]. A decrease in φ_P_ accompanied by a reduction in τ_p_ by an order of magnitude was also observed for the Zn-EtioP-I molecule in a mixture of solvents with the addition of ethyl iodide in [20]. Data on probabilities of ISC for Zn-Etio-I led authors [20] to a conclusion that negative sign of heavy atom effect could be due to the redistribution of the probabilities of the radiative T_1_→S_0_ and non-radiative T_1_~~>S_0_ transitions, resulting in an increase in the probability of the latter process.

As seen from Table 1, the sum of φ_F_ and φ_P_ is much less than unity. Therefore, there exist nonradiative transitions, the probability of which exceeds that of radiative ones. Additionally, the minimum yield formation of triplets can roughly be assessed by the quantum yield of singlet oxygen generation (φ_Δ_), and the sum of φ_Δ_ and φ_F_ from Table 1 is also much less than unity, too. Therefore, rest of the energy is lost during relaxation from the singlet state through nonradiative S_1_~~>S_0_ transitions. It was found out that InCl-EtioP-I generates singlet oxygen ^1^O_2_(^1^Δ_g_) more efficiently in pure toluene (φ_Δ_ = 81%) than in the mixed solutions (φ_Δ_ ≈ 60%).

Average fluorescence durations<τ>_F_ calculated for two exponential curves and energy data φ_F_ were used to calculate the probability of radiative transition *k*_F_. Both in toluene and its mixtures, the value of *k*_F_ varies within a relatively narrow range of 0.6–2.4 × 10^6^ s^−1^, which does not contradict the data for Zn-EtioP-I [26]. Low values of *k*_F_ indicate the presence of a quasi-forbidden long-wavelength transition in the InCl-EtioP-I molecule, which explains the observed higher intensity of the 0-0 fluorescence band with respect to the vibronic bands. There is a noticeable discrepancy between the *k*_F_ values calculated from experimental data and the corresponding values obtained from quantum chemical calculations. 

Generally, high value of φ_Δ_, intensive phosphorescence at 77K and low value of φ_F_ at 298 K suggest that after the absorption of a photon the InCl-EtioP-I complex with a high probability goes into the long-lived triplet T_1_-state, and the preferred way of its relaxation is the energy transfer to oxygen. Therefore, from the viewpoint of the photophysical properties of an individual molecule, InCl-EtioP-I is a suitable electron donor material for organic photovoltaics [13].

## 3. Materials and Methods

InCl-EtioP-I was synthesized using the method described in [12]. Toluene and mixtures based on it, vitrifying at liquid nitrogen temperatures, were used as solvents; for example, toluene:diethyl ether at 1:2 vol. ratio and toluene:diethylether:methyl iodide at 1:2:1 vol. ratio. Toluene dissolves InCl-EtioP-I well and, when mixed with diethyl ether in a ratio of 1:2, forms a glassy matrix upon freezing to 77 K, which is necessary for carrying out relevant spectral and photophysical studies. It was assumed that the addition of methyl iodide does not practically deteriorate the quality of the frozen transparent matrix, but increases the intensity of the phosphorescence [4,27], which is the manifestation of the external heavy atom effect. The solutions were frozen in cryostat with a quartz Dewar vessel, where the samples were fully immersed in the liquid nitrogen.

### 3.1. Spectral Measurements

Electronic absorption spectra were measured using a Cary 500 spectrophotometer (Varian, Australia). Spectra of luminescence (fluorescence, phosphorescence) and excitation spectra of luminescence were measured on an upgraded fluorimeter SDL-2 (LOMO), consisting of an MDR-12 high-aperture excitation monochromator and an MDR-23 registration monochromator. A xenon short-arc lamp XBO-150W/1 (OSRAM) was used as an excitation source. Fluorescence was detected by the cooled photomultipliers FEU-100 (230–800 nm) and FEU-62 (600–1100 nm) operating in the photon-counting mode [27].

Measurements of the fluorescence kinetics and luminescence spectra were carried out on a multifunctional spectrofluorimeter Fluorolog-3 (Horiba Scientific, Japan) with T-channel optics and a pulsed excitation source LDH-D-C-375 (laser diode, λ_ex._ = 376 nm, Δ*t*_1/2_ ≈ 300 ps, *f* = 10 MHz). The decay curve parameters (characteristic decay times and relative integrated luminescence intensities) were calculated using a DAS6 program by Horiba Scientific (see Ref. [27] for more details).

### 3.2. Photophysical Measurements

The kinetics of phosphorescence decay were recorded by the pulse method using an MDR-2 monochromator, an FEU-83 photomultiplier, and a BORDO 221 digital oscilloscope operating in the averaging mode for 100 measurement cycles. The excitation source was the second harmonic of a pulsed neodymium laser LS-2131M with λ_ex_ = 532 nm, Δ*t*_1/2_ ≈ 8 ns, *f* = 4–10 Hz and pulse energy of ~20–30 mJ (LOTIS TII). The phosphorescence decay curve *I*(t)was displayed on a log scale, the phosphorescence decay time τ_P_ was derived from the slope of the logarithmic time dependence.

Quantum yields of fluorescence (φ_F_) and singlet oxygen of luminescence (φ_Δ_) at room temperature were determined by the relative method with toluene solutions of tetraazaporphin (φ_F_ = 0.18) [28] and Pt-dibenzo-tetraaza-iso-bacteriochlorin (φ_Δ_ = 0.72) [29] as standards, respectively. The phosphorescence quantum yields φ_P_ at 77 K were calculated relative to the fluorescence quantum yield φ_F_ using the ratio of the areas under the fluorescence and phosphorescence spectral curves.

### 3.3. Quantum Chemistry 

The ground state geometry of the InCl-EtioP-I molecule was optimized at the DFT level using the B3LYP functional in combination with the def2-TZVP basis set [30]. A pseudopotential combined with the corresponding basis set were used to obtain the core electron shells of the indium atom. We used the multiconfiguration Dirac–Hartree–Fock-adjusted pseudopotential for describing the doubly occupied 1s, 2s, 2p, 3s, 3p and 3d orbitals were described by [31]. The calculations were performed using Orca 5.0.3 [32]. A structure having a *C*_4_ symmetry has been obtained, in which the ligand has a non-planar conformation with a doming-distorted tetrapyrrole skeleton [12]. Hessian calculations indicate the absence of imaginary vibrational frequencies and, hence, the optimized structures correspond to the minima on the potential energy surface. Vibronically resolved fluorescence (from S_1_ state) spectra at 77 K and 298 K were calculated by the path integral approach implemented in the ESD subprogram of Orca. The fluorescence spectra were modeled using Lorentz functions with a linewidth of 50 cm^−1^.

## 4. Conclusions

Spectral luminescence and photophysical properties of a new etioporphyrin-I complex with indium(III) chloride in toluene and its mixtures with diethyl ether and methyl iodide were experimentally studied. The additionally performed quantum-chemical calculations predict several vibronic transitions which might influence the intensity of the main emission band, but do not fully correlate with the experimental data.

At liquid nitrogen temperatures (77 K), in a mixture of toluene with diethyl ether in a ratio of 1:2 a high quantum yield of phosphorescence (10.2%) and a long decay time (17 ms) are observed. Under the same conditions, the ratio of the integral phosphorescence-to-fluorescence intensity is equal to 26.1. This is a record value among other metal complexes of etioporphyrin-I, indicating that the process of the population of the T_1_ state in InCl-EtioP-I is very effective. Formation and relaxation pathways of the T_1_ play are important to understand the photoresponse in active (solar cells) and passive (photodetectors) electronic devices employing small molecules as thin-film semiconductors or photosensitizers [12,13,30,31].

The external heavy atom effect associated with the addition of methyl iodide to a solution compensates for the contribution to the spin–orbit interaction made by the insertion of indium in the porphyrin window (internal effect), i.e., there is a destructive interference of spin–orbit perturbations.

At room temperatures (298 K), the efficiency of the population of the triplet T_1_ state in the InCl-EtioP-I molecule and the predominant pathway of its relaxation through energy transfer is confirmed by the high quantum yield of singlet oxygen formation (~60%). Therefore, the photophysical properties, combined with sublimation and film-forming ability [12], make InCl-EtioP-I a promising material for organic optoelectronics.

## Figures and Tables

**Table 1:** Selected photophysical parameters of InCl-EtioP-I molecule.

Solvent	T, K.	τ_F_, ns *		φ_F_, %	I_P_/I_F_	φ_P_, %	φ_Δ_, %	τ_p_, ms
		τ_1_, ns/B_1_, %	τ_2_, ns/B_2_, %					
Toluene	298	0.19/96.5	3.31/3.5	0.33	-	-	81	-
toluene + diethyl ether (1:2)	298	0.21/51.8	1.63/48.2	0.30	-	-	58	-
	77	0.42/34.2	2.19/65.8	0.39	26.1	10.2	-	17.0
toluene + diethyl ether + CH_3_I (1:2:1)	298	0.13/74.2	3.77/25.8	0.19	-	-	62	-
	77	0.25/74.4	4.23/25.6	0.37	19.2	7.1	-	6.0

* characteristic decay times (τ_1_, τ_2_) and relative integrated intensities (B_1_, B_2_) for two-exponential fluorescence.

## Data Availability

Research data are available upon request.
